# Frontal Alpha Asymmetry and Inhibitory Control among Individuals with Cannabis Use Disorders

**DOI:** 10.3390/brainsci9090219

**Published:** 2019-08-29

**Authors:** Alina Shevorykin, Lesia M. Ruglass, Robert D. Melara

**Affiliations:** 1 Pace University, Department of Mental Health Counseling and Psychology, 861 Bedford Road, Marks 22, Pleasantville, NY 10570, USA; 2 Rutgers University, Center of Alcohol and Substance Use Studies, Graduate School of Applied and Professional Psychology, 607 Allison Road, Smithers Hall, 222, Piscataway, NJ 08854, USA; 3 The City College of New York, CUNY, Department of Psychology, 160 Convent Avenue, NAC 7120, New York, NY 10031, USA

**Keywords:** cannabis use disorder, cue reactivity, craving, inhibitory control, frontal alpha asymmetry, EEG, cannabinoids

## Abstract

To better understand the biopsychosocial mechanisms associated with development and maintenance of cannabis use disorder (CUD), we examined frontal alpha asymmetry (FAA) as a measure of approach bias and inhibitory control in cannabis users versus healthy nonusers. We investigated: (1) whether FAA could distinguish cannabis users from healthy controls; (2) whether there are cue-specific FAA effects in cannabis users versus controls; and (3) the time course of cue-specific approach motivation and inhibitory control processes. EEG data were analyzed from forty participants (CUD (*n* = 20) and controls (*n* = 20)) who completed a modified visual attention task. Results showed controls exhibited greater relative right hemisphere activation (indicating avoidance/withdrawal motivation) when exposed to cannabis cues during the filtering task. By contrast, cannabis users exhibited greater relative left activation (approach) to all cues (cannabis, positive, negative, and neutral), reflecting a generalized approach motivational tendency, particularly during later stages of inhibitory control processes. The difference between cannabis users and controls in FAA was largest during mid- to late processing stages of all cues, indicating greater approach motivation during later stages of information processing among cannabis users. Findings suggest FAA may distinguish cannabis users from healthy controls and shows promise as a measure of inhibitory control processes in cannabis users.

## 1. Introduction

Among individuals age 12 and older, cannabis is the most widely used illicit drug, with an estimated 24 million people reporting past month use in 2016 [1]. The largest increases from 2002 to 2016 were among adults age 26 and older [1]. Studies suggest an increase in permissive attitudes towards cannabis use and reduced perceptions of cannabis-related harm may underlie increases in cannabis use among adults [2,3]. However, studies also show that both short-term and long-term/chronic cannabis use are associated with detrimental psychological and physical effects. For example, acute negative effects may include impairments in attention, short-term memory, and motor coordination, increasing risk for accidental injuries [4,5]. Long-term or heavy use of cannabis may increase the risk for developing a cannabis use disorder (CUD) [6], which may contribute to lasting structural and functional brain changes. The potency of cannabis has also been increasing significantly over the past decade and serves as a significant risk factor for the onset of CUD symptoms [7,8]. Data from two waves of the National Epidemiological Survey on Alcohol and Related Conditions [9] revealed that, among cannabis users, three out of 10 evidenced a Diagnostic and Statistical Manual of Mental Disorders, 4th Edition (DSM-IV) past-year CUD, with rates of CUD doubling from 2001–2002 to 2012–2013. Given these increases and associated consequences, there is continued need to better understand the underlying processes associated with the development and maintenance of CUD, which is critical to the development of novel interventions [2]. 

Evidence suggests there are several key brain-based mechanisms involved in the maintenance of CUD, including drug cue reactivity, attentional bias, craving, and inhibitory control deficits—all of which underlie drug seeking, consumption, and relapse among those trying to abstain [10,11]. The dual process theory of addictive behaviors integrates these mechanisms by arguing that the approach (appetitive) system and self-regulatory (executive function/control) systems become imbalanced throughout the addiction process [12,13,14]. The approach-oriented (appetitive) system underlies the automatic behavioral tendency to approach one’s drug of choice (i.e., approach bias). This automatic approach tendency is presumed to be a function of sensitization that occurs as a result of repeated and persistent drug use contributing to a heightened response to drugs and conditioned drug cues [15,16]. In tandem, a deficit in the executive control system, and reduced inhibitory control in particular, makes it difficult to resist or inhibit the impulse to approach and use drugs [13,14,17].

Over the last several decades, an emerging body of research has positioned frontal alpha asymmetry (FAA), measured via electroencephalogram (EEG), as a promising neural index of the approach motivational system [18,19,20]. FAA is the difference between left and right alpha activity/activation in the frontal cortical areas of the brain. FAA has been widely studied as a psychophysiological index in research on motivation, cognition, and psychopathology [21,22,23,24]. Alpha activity is cortical EEG activity in the alpha frequency band (8–13 Hz) recorded over a period of time and is believed to reflect an individual’s tendency or predisposition to engage in certain motivational or emotional responses [25]. Alpha activation, on the other hand, is a task-related change in alpha activity [25], which has been investigated in relationship to current emotions and behaviors [26,27]. 

The approach/withdrawal motivation model of EEG asymmetry suggests that greater relative left frontal activity is associated with approach-related tendencies, and greater relative right activity is associated with withdrawal-related tendencies [28]. A majority of the studies to date have examined the link between FAA and various psychopathological conditions, including depression [21,29,30,31] and ADHD [32,33]. Overall, these studies lend support to the motivational theory that both a behavioral inhibition system (BIS) and a behavioral activation system (BAS) drive our emotions and behaviors. Evidence suggests that a novel/aversive stimulus results in the organization of cognitive resources for removal or rejection of the stimulus (i.e., behavioral inhibition), while an incentive/appetitive stimulus results in the organization of cognitive resources to attain the desired stimulus (i.e., behavioral approach) [34].

Despite its relevance for understanding addictive behaviors, there has been a dearth of studies examining FAA among those who use alcohol/drugs or have a substance use disorder. In one of the few studies examining FAA among substance using individuals, Gable and colleagues [35], in a nonclinical sample of 42 college students who reported alcohol use in the past month, found that, among those with high impulsivity, there was relatively greater left frontal alpha asymmetry in response to alcohol cues. The authors speculated that the inhibitory control system may serve a regulatory function in the neural response to alcohol cues. Likewise, Bowley and colleagues [36] found a trend among college students for left frontal activity enhancement after exposure to beer stimuli, suggesting enhanced approach motivation [36]. Others have also investigated the role of FAA in areas such as attentional narrowing and inhibitory control in alcohol-related cognitions [35], as well as craving and cue reactivity in nicotine dependence [37]. A majority of these studies, however, were conducted with college students with varying levels of alcohol use or with individuals with nicotine dependence. Thus, the findings may not be generalizable to those with other substance use or use disorders such as CUD. To our knowledge, there have been no studies to date that have examined FAA among those with CUD. Moreover, the studies reviewed have all examined FAA averaged over time by stimulus; it thus remains unclear whether approach motivation and inhibitory control processes are static or shifting over time during the course of information processing [32]. 

The aims of our study were thus threefold: (1) to examine whether FAA could distinguish individuals with CUD from healthy controls; (2) to examine if there are cue-specific FAA effects in cannabis users versus healthy controls; and (3) to determine the time course of approach motivation and inhibitory control processes during processing of cannabis cues. Given the dearth of studies examining the processes underlying CUD, this study represents a novel and important area of research, with potential implications for our understanding of the neurobiological mechanisms of CUD with implications for development of treatment interventions.

## 2. Materials and Methods

Data for this analysis came from a recently completed lab-based experimental study that utilized EEG and ERP (event-related potential) to examine the time course of attentional bias and cue reactivity among individuals with CUD compared to healthy controls. See Ruglass et al. [38] for details. The parent study examined ERPs as indices of attentional bias to cannabis cues in cannabis users. By contrast, the current study leveraged the EEG data collected to examine FAA as a measure of approach motivation in cannabis users.

### 2.1. Participants

Forty participants were recruited from printed flyers, online advertisements, and word of mouth (see Table 1 for demographic characteristics). For full details on inclusion and exclusion criteria, see Ruglass et al. [38]. The current study included cannabis smokers (*n* = 20, M_age_ = 26.2, SD = 8.53) who were physically healthy English-speaking adults and were diagnosed with current CUD (abuse or dependence). Similarly, the study included healthy controls (*n* = 20, M_age_ = 28, SD = 10.87) who were physically healthy English-speaking adults and did not meet criteria for any current or past psychiatric or substance use disorders according to the DSM-IV [39]. Participants in the CUD group were excluded if they had any other current or past psychiatric disorder, or a positive drug test for any substance other than cannabis. Participants were excluded from the healthy control group if they had a positive drug test for any drug. Participants were also excluded from the study if they reported suicidality or homicidality, history of seizures, organic mental syndrome, or they refused to be audio recorded. All participants self-reported normal or corrected normal visual acuity. Informed consent was obtained from each participant. The Institutional Review Board of the City University of New York reviewed and approved all materials and procedures. Participants were compensated $100 in cash for their time, effort, and transportation for both sessions.

### 2.2. Materials and Procedure

**Figure 1:**
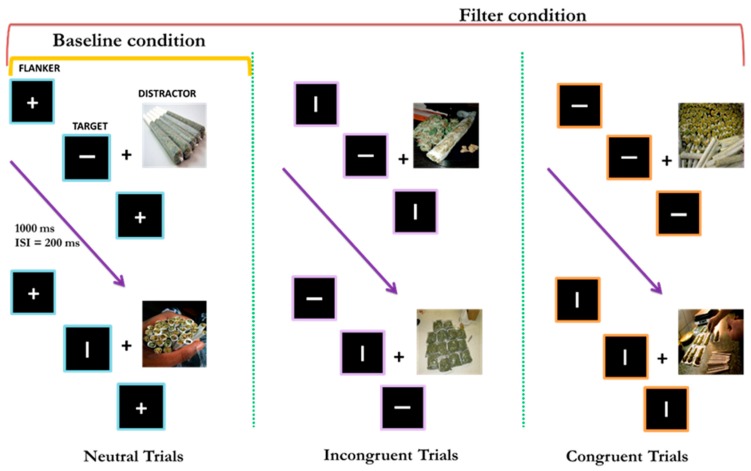
Modified flanker task, made up of a block of 80 trials, each consisting of a fixation square followed by the first flanker, target, and second flanker (stimulus displays), presented sequentially for 150 ms separated by a random interstimulus interval (153–390 ms). The target was represented by a vertical or horizontal line, or a cross superimposed on a cannabis-related picture or a neutral, positive, or negative image from the International Affective Picture System (IAPS) [42].

After completing a phone screen, participants completed a baseline interview and an experimental session on two separate days. Participants first completed a urine toxicology screen and were administered a series of diagnostic and clinical measures during both sessions (see Ruglass et al. [38] for details). During the experimental session, while inside an electrically and acoustically shielded Industrial Acoustics Company (New York, USA) chamber, each participant completed 24 blocks of experimental trials (and one or more blocks of practice trials) in a modified version of the visual flanker task [40] called the temporal flanker paradigm (see Figure 1) while their electroencephalographic (EEG) responses were recorded. The visual flanker task is a traditional cognitive method for measuring the extent to which distractor stimuli draw attention away from and affect the processing of target stimuli [41]. The current study investigated whether there is a difference in attentional control to various cues between cannabis users and nonusers as measured by FAA elicited in a version of the visual flanker task called the temporal flanker paradigm. The experimental session lasted approximately 3 hours, including EEG preparation and short breaks.

Stimuli were created in Presentation® (Neurobehavioral Systems). Each task in the visual flanker paradigm was made up of a block of 80 trials, each consisting of a fixation square (0.67º) followed by a first flanker, target, and second flanker (stimulus displays), presented sequentially for 150 ms separated by a random interstimulus interval (153–390 ms), see Figure 1. The target was represented by a vertical or horizontal line, or a cross superimposed on one of the following images: a cannabis-related picture, or a colored picture from the International Affective Picture System (IAPS) [42] that was pre-judged to be neutral (e.g., door scene), positive (e.g., beach scene), or negative (e.g., natural disaster scene). IAPS pictures’ valence and arousal ratings range from 1 (low pleasure or low arousal, respectively) to 9 (high pleasure or high arousal, respectively). Neutral images were considered those with a mean rating of five. Average valence/arousal of IAPS stimuli included in this study were 5.20/3.07 for neutral cues, 7.23/5.24 for positive cues, and 2.81/5.60 for negative cues. Cannabis-related pictures were collected from free online sources and included images of cannabis, joints, and cannabis paraphernalia (e.g., pipe, rolling paper). Cannabis pictures were matched with IAPS pictures in terms of size. Line stimuli, subtending a visual angle of 0.57º, appeared in gray on a black background; cues subtended 10.85º (V) × 9.74º (H) of the visual angle.

Overall, each participant completed 24 blocks of trials, consisting of 3 repeating sets of 8 blocks: 4 baseline and 4 filtering for each image type (neutral, positive, negative, and cannabis). The order of blocks was balanced across participants. Each block consisted of 80 trials, in which participants were asked to respond by clicking the mouse key as quickly and accurately as possible to the orientation of the target line, while ignoring the flanker lines and the surrounding image. Assignment of line orientation to response keys was counterbalanced across participants, and tasks were divided into baseline and filtering [43]. 

In the baseline task, distractors were held constant across trials, such that two crosses always flanking the target and a single image (cue), drawn from one of four cue sets (neutral, positive, negative, and cannabis), appearing on each trial in a block. Distractors were held constant in order to create a baseline measurement of attention with minimal distraction. Participants immediately repeated the task if they did not reach the required 80% level of accuracy for the baseline condition. For the filtering task, distractors appeared randomly, such that target and flanker lines matched in terms of direction (congruent trials) in 40% of trials (32 of 80) and did not match in terms of direction (incongruent trials) in 40% of trials (32 of 80), while in 20% of trials (16 of 80), the flankers were crosses (neutral trials). The use of congruent and incongruent distractors in the filtering task introduces stimulus conflict as an attentional requirement of the task, thereby creating a measurement of inhibitory control when compared to participants’ baseline performance. Participants immediately repeated the task if they did not reach the required 60% level of accuracy in the filtering condition. All eight images from one of the four cue sets (neutral, positive, negative, cannabis) appeared randomly an equal number of times during each block of filtering trials, creating four distinct filtering tasks. See Ruglass et al. [38] for further details.

### 2.3. Data Recording and Analysis

EEG recordings were collected, using an ANT neuro system (ANT, Philadelphia, PA, USA) in a high-density (128 electrodes) montage arranged in a cap, continuously at a sampling rate of 512 Hz. An electrooculogram (EOG) was used to monitor blinks and other eye movements from two electrode montages, one electrode was placed on the infra- and supra-orbital ridges of the right eye (VEOG), and the other was placed on the outer canthi of each eye (HEOG). Trials in which mastoid activity was greater than 100 μV were excluded. Trials with excessive blinks, eye movements, or other movement artifacts were defined as z-values on the VEOG, HEOG, and lowermost scalp channels exceeding 4.5 in a frequency band between 1 and 140 Hz; a MATLAB routine [44] was used to remove artifact trials.

Stimulus-locked waveforms (sweep time = 2000 ms) were referenced to linked mastoids band-pass filtered between 0.1 and 30 Hz. Induced alpha power (8–13 Hz) was extracted using a Morlet wavelet transform (spectral bandwidth = 6–8 Hz; wavelet duration = 80–106 ms) individually on each trial of each task (baseline, filtering) to each cue (neutral, cannabis, positive, negative) in each of three time epochs (0–200, 201–400, and 401–800 ms), separately for each of three pairs of lateral electrode locations: frontal (F7, F8), midfrontal (F3, F4), and midline frontocentral (FC3, FC4). Alpha power was log transformed [45] to derive a composite measure of FAA [25,46,47], as follows:

FAA = (ln[αF8] + ln[αF4] + ln[αFC4])/3 − (ln[αF7] + ln[αF3] + ln[αFC3])/3(1)

where α is induced alpha power at the corresponding frontal electrode locations. Higher FAA scores indicate relatively higher left cortical activity [48]. 

We performed mixed model analyses of variance (ANOVAs) on FAA scores using Statistica® software, with Group (2 levels: cannabis smokers, healthy controls) as the between-subjects factor and Task (2 levels: baseline, filtering), Cue (4 levels: neutral, cannabis, positive, negative), Frequency (6 levels: 8–13 Hz), and Epoch (3 levels: early, middle, and late) as within-subject factors. To guard against violations of the sphericity assumption with repeated-measures data, all main effects and interactions reported as significant were reliable after Greenhouse–Geisser correction [49]. 

## 3. Results

**Figure 2:**
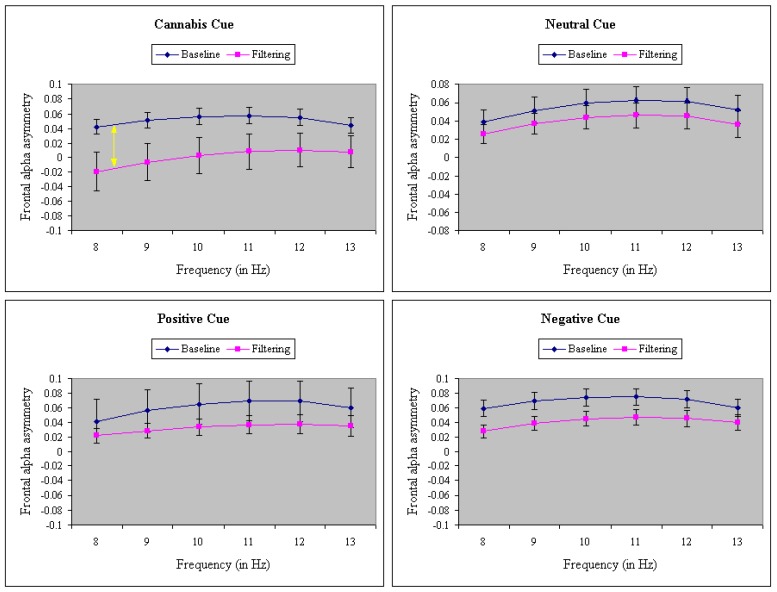
A significant three-way interaction of task (baseline vs. filtering), cue (cannabis, neutral, positive, and negative) and frequency (8–13 Hz) on frontal alpha asymmetry. Larger task difference at low alpha was especially prominent to cannabis cues relative to neutral, positive, and negative cues. Error bars represent the standard error of the mean.

ANOVA of FAA uncovered a significant main effect of Task, F(1,38) = 19.00, *p* < 0.001, MSe = 0.07, η^2^ = 0.22. FAA was significantly more positive in the baseline task (0.06) than in the filtering task (0.03). There was also a main effect of Frequency, F(5,190) = 9.07, *p* < 0.001, MSe = 0.01, η^2^ = 0.05. Post-hoc analysis (Newman–Keuls, 0.05 criterion level) revealed less left-sided activation at 8 Hz (low alpha) than at the other five alpha frequencies. Moreover, the difference in FAA between baseline and filtering tasks was larger at lower alpha (8–9 Hz) than higher alpha (12–13 Hz), F(5,190) = 2.52, *p* = 0.03, MSe = 0.001, η^2^ = 0.002, particularly at the later time epochs, F(10,380) = 7.75, *p* < 0.001, MSe = 0.0002, η^2^ = 0.003. As shown in Figure 2, the larger task difference at low alpha was especially prominent to cannabis cues relative to neutral, positive, and negative cues, leading to a significant Task x Cue x Frequency interaction F(15,570) = 2.63, *p* < 0.001, MSe = 0.001, η^2^ = 0.002. Furthermore, the effect of cannabis cues on low alpha during the filtering task was evident only in nonusers, creating a Group × Task × Cue × Frequency interaction, F(15,570) = 1.92, *p* = 0.02, MSe = 0.001, η^2^ = 0.003.

**Figure 3:**
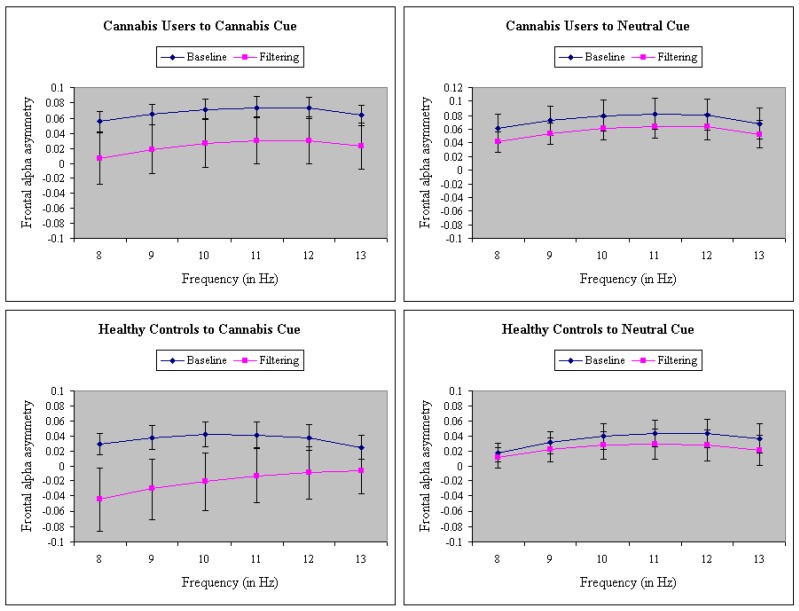
A significant four-way interaction in group (cannabis users vs. controls), task (baseline vs. filtering), cues (cannabis vs. neutral) and frequency (8–13 Hz) on frontal alpha asymmetry. Control participants show right-sided hemisphere (avoidance) frontal alpha asymmetry (FAA) activation only to cannabis cues during the filtering task, and they show left-sided (positive) activation in the other conditions, while cannabis users show left-sided activation (approach) in all conditions. Error bars represent the standard error of the mean.

**Figure 4:**
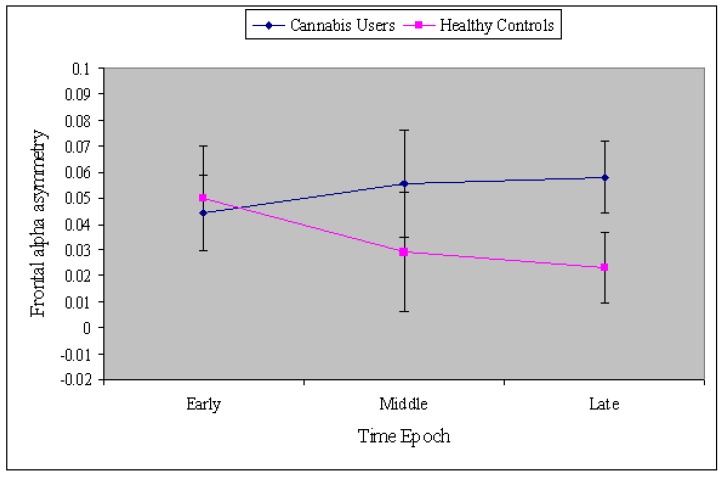
Group difference (cannabis users vs. controls) in FAA. The largest group difference was during the middle and late time epochs. Error bars represent the standard error of the mean. See the online article for the color version of this figure.

Figure 3 depicts the four-way interaction in a pairing of the neutral and cannabis cue conditions only. As one can see, only to cannabis cues in control participants during filtering does FAA show right-sided hemisphere (avoidance) activation. Control participants show left-sided (approach) activation in the neutral, positive, and negative conditions, while cannabis users show left-sided activation (approach) in all conditions (neutral, positive, negative, and cannabis). As shown in Figure 4, the group difference in FAA was largest during the middle (201–400 ms) and late time epochs (401–800 ms), F(2,76) = 3.23, *p <* 0.05, MSe = 0.07, η^2^ = 0.07.

## 4. Discussion

The current study examined three related questions: (1) whether FAA could distinguish individuals with cannabis use disorders from healthy controls; (2) whether there were cue-specific FAA effects; and (3) when in the time course after cue presentation (early, middle, or late) the differences in FAA (an index of approach motivation and inhibitory control processes) are greatest between individuals with cannabis users and healthy controls.

Results revealed that the healthy controls/nonusers exhibited greater relative right hemisphere activation (typically indicative of avoidance/withdrawal motivation; Davidson, 1993; Davidson et al., 1990) when exposed to cannabis cues during the filtering task, especially during early frequencies. By contrast, cannabis users showed greater relative left activation (indicative of approach motivation) during all conditions (neutral, positive, negative, and cannabis). It is possible that healthy controls withdraw their attention to potent (cannabis) distractors as a way to enhance inhibitory control and improve performance. Healthy controls may also perceive cannabis cues as unpleasant or aversive (particularly when there are multiple cannabis cues being presented randomly), triggering the withdrawal/avoidance system. These findings are consistent with the theory of behavioral inhibition system (BIS) [34] and may reflect participants’ organization of cognitive resources for avoidance of aversive stimuli. 

Counter to expectations, the cannabis cues did not elicit greater approach motivation among cannabis users. Instead, cannabis users exhibited greater relative left hemisphere activation (approach) across all conditions (neutral, positive, negative, and cannabis and neutral cues), suggesting positive feelings and/or high engagement with all stimuli. Cannabis users also evidenced higher FAA (greater relative left activation) compared to the healthy controls during the middle and late stages of processing of all cues, reflecting a generalized approach motivational tendency, particularly during the later stages of inhibitory control processes. It is possible that, due to an altered reward processing system secondary to structural (particularly in the prefrontal cortex) and functional changes in the brain as a result of long-standing and continual cannabis use [50], the cues elicited a generalized approach tendency among cannabis users. 

Conversely, it is possible that the cannabis cues were not salient enough for the cannabis users—particularly during the latter phases of cue processing where more consciously controlled inhibitory processes are at play—to activate greater approach-related tendencies above and beyond those activated for the neutral, positive, or negative cues. It is also possible that the neutral cues were not perceived as “neutral” by our CUD participants, contributing to similar levels of left activation. Indeed, FAA may index both motivational (approach versus avoid) and affective processes (positive or negative) [18]. Thus, cannabis users’ greater relative left activation to all cues suggests more positive feelings and greater engagement with all stimuli. Studies also suggest that individual differences in substance use duration and severity and trait impulsivity may play important roles in the degree to which there is greater left activation to appetitive cues (e.g., substance use cues) compared to neutral cues [51,52,53]. For example, Mechin and colleagues (2016) found a positive correlation between trait impulsivity and left activation to alcohol cues, even after controlling for recent drinking behaviors, highlighting the importance of examining individual difference variables. Future studies that examine personality differences such as impulsivity, among cannabis users and level of severity of cannabis use disorder and their influence on FAA are critical to further understand FAA processes among this population. 

### Limitations

A few limitations should be mentioned. The generalizability of our findings is limited to nontreatment-seeking individuals with CUD. Moreover, our small sample size may have limited our ability to detect significant effects among cannabis users. Future research is needed with a larger sample size and follow-up timepoints for replication of findings, examination of individual differences in the associations between cue exposure and FAA, as well as determination of whether greater relative left FAA predicts increased craving and future cannabis use among those with cannabis use disorders.

## 5. Conclusions

Despite limitations, this study is one of the first to investigate FAA among those with cannabis use disorder and contributes to the growing literature on the relationship between FAA and motivational or inhibitory control processes, especially in substance users, by highlighting the usefulness of FAA as a measure of motivational processes. Methodological strengths include the careful matching of controls to cannabis users, the lack of psychiatric and other drug use comorbidities, biomedical verification of cannabis and other substance use, as well as measurement of neural activity via EEG. Results suggest that FAA holds significant promise as a measure of attentional and motivational processes in cannabis users, with promising areas of future research, including utilizing FAA as a transdiagnostic marker that distinguishes cannabis users from healthy controls; FAA as a measure or mechanism of cue reactivity and specifically the impact of cues on attention; and the potential for attentional or approach-bias modification training to influence motivation and inhibitory control processes in substance users.

## Figures and Tables

**Table 1:** Demographic characteristics.

Variable	CUD (*n* = 20) M (SD) or %	Controls (*n* = 20) M (SD) or %	t statistics/χ^2^
**Baseline Session**			
Age	26.2 (8.53)	28 (10.87)	*t* = 0.58, *p =* 0.56
Education^1^	13.85 (1.63)	14.85 (1.35)	*t* = 2.11, *p = 0*.041
Sex (% males)	80%	75%	χ^2^ = 0.143, *p =* 0.705
Marital Status (% Single)	100% (*n* = 20)	95% (*n* = 19)	χ^2^ = 2.105, *p =* 0.35
**Race/Ethnicity**			
Black/African American	50% (*n* = 10)	45% (*n* = 9)	χ^2^ = 1.26, *p =* 0.74
Hispanic/Latino	30% (*n* = 6)	25% (*n* = 5)	
White	20% (*n* = 4)	25% (*n* = 5)	
Other	0% (*n* = 0)	5% (*n* = 1)	
**Employment**			
Full-time	45% (*n* = 9)	25% (*n* = 5)	χ^2^ = 6.56, *p* = 0.161
Part-time	30% (*n* = 6)	50% (*n* = 10)	
Student	15% (*n* = 3)	25% (*n* = 5)	
Unemployed	10% (*n* = 2)	0%	
**Cannabis Use**			
Past week use of cannabis (# of days)	6.4 (1.85)	N/A	N/A
Past 90 days use of cannabis (# of joints)	246.1 (183.35)	N/A	N/A

^1^*p* < 0.05.
